# Biomechanical Behavior of Injected Cement Spacers versus Traditional Cages in Low-Density Lumbar Spine under Compression Loading

**DOI:** 10.3390/medicina60071155

**Published:** 2024-07-17

**Authors:** Tibor Csakany, Peter Varga, Boyko Gueorguiev, Eva Lakatos, Marta Kurutz

**Affiliations:** 1 National Center for Spinal Disorders, 1126 Budapest, Hungary; 2 AO Research Institute Davos, 7270 Davos, Switzerland; 3 Department of Structural Mechanics, Faculty of Civil Engineering, Budapest University of Technology and Economics, 1111 Budapest, Hungarykurutzm@eik.bme.hu (M.K.)

**Keywords:** lumbar stabilization, osteoporosis, injected cement spacers, traditional cages, bone–implant interface, biomechanical compression test

## Abstract

*Background and Objectives*: Osteoporosis renders the use of traditional interbody cages potentially dangerous given the high risk of damage in the bone–implant interface. Instead, injected cement spacers can be applied as interbody devices; however, this technique has been mainly used in cervical spine surgery. This study aimed at investigating the biomechanical behavior of cement spacers versus traditional cages in lumbar spine surgery. *Materials and Methods*: Destructive monotonic axial compression testing was performed on 20 human cadaveric low-density lumbar segments from elderly donors (14 f/6 m, 70.3 ± 12.0 y) treated with either injected cement spacers (*n* = 10) or traditional cages (*n* = 10) without posterior instrumentation. Stiffness, failure load and displacement were compared. The effects of bone density, vertebral geometry and spacer contact area were evaluated. *Results*: Cement spacers demonstrated higher stiffness, significantly smaller displacement (*p* < 0.001) and a similar failure load compared to traditional cages. In the cage group, stiffness and failure load depended strongly on bone density and vertebral height, whereas failure displacement depended on vertebral anterior height. No such correlations were identified with cement spacers. *Conclusions*: Cement spacers used in lumbar interbody stabilization provided similar compression strength, significantly smaller failure displacement and a stiffer construct than traditional cages that provided benefits mainly for large and strong vertebrae. Cement stabilization was less sensitive to density and could be more beneficial also for segments with smaller and less dense vertebrae. In contrast to the injection of cement spacers, the optimal insertion of cages into the irregular intervertebral space is challenging and risks damaging bone. Further studies are required to corroborate these findings and the treatment selection thresholds.

## 1. Introduction

Degenerative changes of intervertebral discs are generally accompanied by osteoporosis, rendering the required surgical interventions dangerous. The decreased bone density and endplate deformity make the fitting of interbody devices in the intervertebral space imperfect and may result in large spacer subsidence and damage in the bone–implant interface leading to a loss of stability. Biomechanical [1,2,3,4,5,6], numerical [7,8] and clinical [9,10,11] studies and reviews [12] have reported subsidence and stability following the use of traditional cages to be very sensitive to bone quality, cage footprint and position, and leading to pain and decreased functional capacity, especially in the case of osteoporosis and traumatic circumstances.

An alternative interbody stabilization method with the use of injected bone cement has been developed for cervical spine surgery [13,14], with studies demonstrating that the replacement of herniated cervical discs by bone cement can be considered as a safe operative procedure [15,16,17,18].

However, despite the successful application of injected polymethylmethacrylate (PMMA) spacers in cervical spine surgery, their use in the lumbar spine has not been widely utilized yet. Injected PMMA spacers have been successfully implemented in open transforaminal lumbar interbody fusion (TLIF) surgeries with posterior stabilization in the National Center for Spinal Disorders in Budapest [19], where a percutaneous surgical method of filling the intervertebral space with PMMA bone cement—referred to as percutaneous cement discoplasty (PCD)—has been recommended as an alternative treatment option for elderly patients with severe discopathy and moderate osteoporosis [20], arousing interest regarding indications/contraindications, disc herniation, osteoporosis, adjacent level fractures and cement leakage. According to relatively short-term clinical studies, PCD is a feasible technique for symptomatic lumbar disc herniation with certain endplate changes [21,22]. Referring to the pioneering study of Varga et al. [19], Sola et al., also introduced the PCD technique, and based on the postoperative imaging results, it was claimed that PCD could be an alternative minimal invasive strategy for the treatment of advanced degenerative disk disease [23], leading to a lively discussion [24,25,26,27]. A retrospective analysis evaluated the effects of PCD on spinopelvic radiological parameters and their associations with clinical outcomes [28], initiating further discussion [29,30,31,32] and concluding that PCD is an alternative method in symptomatic aging spine patients with the main pathology characterized by vertical instability and consequent foraminal stenosis with nerve root irritation. Another retrospective clinical analysis of 54 patients with advanced disc disease with an at least 1-year follow-up showed PCD to be, at least for a short-term follow-up, an effective option in lower-back pain disease [33]. With a minimum 2-year follow-up of 156 patients, another study reported that PCD showed significant improvement after 2-year of follow-up with a relatively low rate of complications [34]. Based on the prospective clinical and radiological data of 10 patients, a novel three-dimensional method was developed to measure geometrical changes in treated spine segments, indicating that surface area and volume of injected PMMA spacers can predict the extent of indirect spinal canal decompression [35].

A biomechanical study in an in vitro model tested ten porcine lumbar spine segments in flexion, extension, lateral bending and compression, concluding that discoplasty recovered intervertebral posterior height by opening the neuroforamen—as clinically observed—with no influence on stiffness or spine mobility [36]. Another recent study developed a biomechanical model of PCD in ovine functional spinal units, concluding that discoplasty treatment could restore disc height and axial stiffness after injury [37]. The effect of cement strength in PCD was investigated with finite element simulations, indicating that discoplasty with low-modulus PMMA could reduce the stresses on the endplates [38]. A finite element analysis study proved that nerve root stress decreased after intervertebral height recovery through cement injection, resulting in a significant indirect decompression effect [39]. Eight fresh pig lumbar spines were tested in intact, post-nucleotomy, and post-discoplasty states in another study [40]. Flexion/extension, lateral bending and axial rotation were induced by pure moments. The range of motion and neutral zone were recorded, concluding that discoplasty helped enhance the stability of the lumbar spine in flexion/extension and lateral bending, while fractures and sliding of bone cement were observed after discoplasty, suggesting that cement movement may cause nerve compression. Another experimental study tested twenty-seven human spinal segments at 50% body weight in flexion and extension [41]. Posterior disc height, range of motion, segment stiffness and strains were measured. The authors concluded that extreme strains on the discs were reduced by PCD. This study supported the clinical observations in terms of recovered disc height close to the foramen, while PCD helped to stabilize the spine in flexion and did not increase the risk of tissue damage in the annulus.

The indications, clinical outcomes and complications of PCD were systematically reviewed by Fusini et al., concluding that PCD provided good clinical results in elderly patients, especially for pain relief [42]. However, the articles included in this review showed a poor methodologic score that could have affected the conclusions. Another systematic review and meta-analysis stated that PCD showed clinically significant improvements in pain and functional disability; however, due to methodological limitations and a high risk of bias, the validity and generalizability of the findings are uncertain [43]. Despite these issues, the results provide preliminary insights into PCD’s potential efficacy and can guide future research to address current limitations. A recent systematic review on the state of the art of lumbar PCD [44] stated that the papers consistently reported that PCD significantly improved the clinical status of the patients and maintained it after two years; however, clinical and biomechanical investigations would help optimize the surgical technique. Nevertheless, the detailed biomechanical effects of filling the lumbar intervertebral space with PMMA cement have not yet been investigated in comparison with other conventional techniques on human models.

Therefore, the aim of this biomechanical study was to evaluate the primary load-bearing capacity of human lumbar spine segments treated with PMMA cement spacers for lumbar fusion in comparison with traditional polyetheretherketone (PEEK) cages, with particular focus on the bone–implant interface. A preliminary report from a pilot study has been previously published [45]. However, the current work presents a more comprehensive biomechanical assessment of PMMA spacers inserted by an open transforaminal approach. The working hypothesis was that injected PMMA spacers may be able to better accommodate irregular vertebral endplates, yielding a larger vertebra–implant interface area and smoother load transmission; consequently, PMMA spacers would provide smaller subsidence with larger stiffness and compression strength compared to traditional PEEK cages. Moreover, we assumed that the position of the inserted cage has an important role in the load transmission; thus, we paid special attention to the behavior of the bone–implant interface with the cage position.

## 2. Materials and Methods

Ethical approval for this study was granted by the Ethical Commission of the Saint James Hospital, Budapest, Hungary (approval number 151/2009). All procedures were performed in line with the local legislative regulations.

### 2.1. Specimen Preparation

Twenty spinal motion segments or functional spinal units (FSUs) without tumors, remarkable deformities or known bone diseases were extracted from fresh frozen cadaveric lumbar spines of 15 human donors after thawing at room temperature. The total ligament system and the zygapophysial joints were preserved. The specimens were randomized by allocating the vertebrae consecutively to two treatment groups: the PEEK cage group (*n* = 10, male/female: 4/6; one T12-L1, four L1-2, four L3-4 and one L4-5, age 70.2 ± 10.6 years (mean ± standard deviation)) and PMMA spacer group (*n* = 10, male/female: 2/8; six L1-2 and four L3-4, age 70.4 ± 13.9 years).

**Figure 1:**
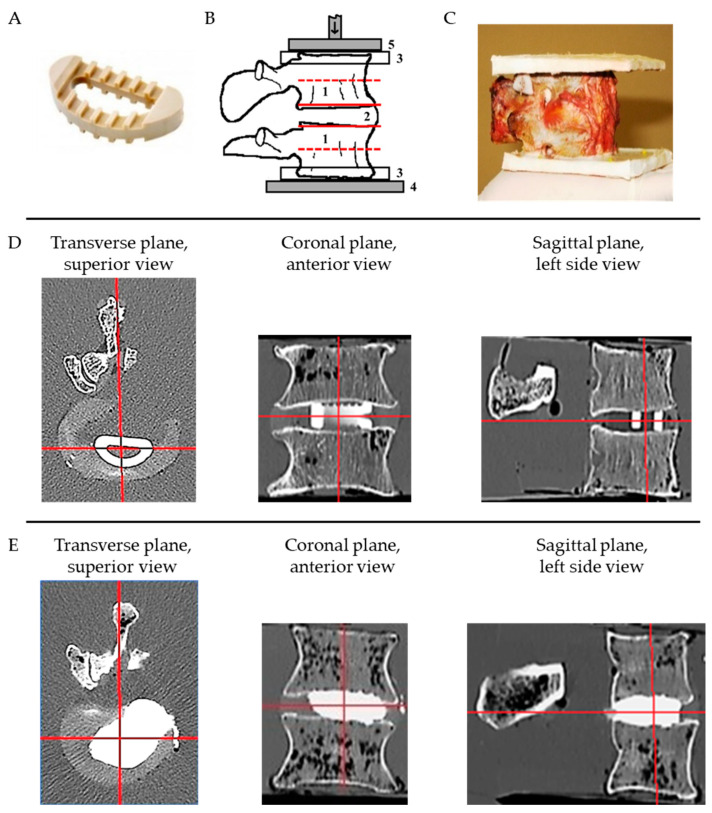
(**A**) PEEK lumbar interbody cage. (**B**) Illustration of a specimen before operation in sagittal view: 1—vertebral bodies, 2—intervertebral space, 3—embedment plates, 4—base plate, 5—head plate of the testing machine, red lines—visualizing subcortical (solid lines) and central levels (dashed lines) for bone mineral density evaluation by CT scanning, arrow—indicating mechanical loading; (**C**) Photograph of a PMMA specimen post treatment in sagittal view. (**D**) Superior transverse, anterior coronal and midsagittal post-operative CT slices of a spacer with a PEEK cage. (**E**) Superior transverse, anterior coronal and midsagittal post-operative CT slices of a spacer with a PMMA spacer.

The free endplates of the cranial and caudal vertebrae of each FSU were embedded in resin (RenCast FC 52/53 DB Isocyanate, RenCast FC 52 BD Polyol, Huntsman Advanced Materials, Basel, Switzerland; Figure 1C). All specimens were submerged in water and scanned by means of computed tomography (CT, Hitachi Presto, Hitachi Medical Corporation, Tokyo, Japan, 120 kVp energy, 150 mA current, 150 ms exposure time, 512 × 512 pixel matrix, 0.47 mm in-plane pixel size, 0.75 mm slice thickness) according to the lumbar spine study protocol (pre-op scans).

Subsequently, each FSU was treated by the same experienced surgeon with either a commercial PEEK thermoplastic D-shaped cage spacer (Sanatmetal, Eger, Hungary; Figure 1A; PEEK group), or using a high-viscosity PMMA bone cement spacer (Cemex Isoplastic, Tecres Medical, Verona, Italy; PMMA group). Both techniques followed the standardized common protocol of the National Center for Spinal Disorders [19]. First, a facetectomy was set on the left side by removing the left articular joint. Following the conventional TLIF surgical technique, a discectomy was performed by creating a window on the annulus and completely removing the nucleus with care to save the endplates’ integrity. In the PEEK group, a cage of medium size (footprint of 320 mm^2^) was placed at the anterior-central part of the intervertebral space, directly behind the anterior annulus (Figure 1D). The constant footprint of the PEEK cage allowed us to focus on the analysis of the contact area fraction effect. PEEK cage heights of either 7, 9, 11 or 12 mm were used to accommodate the height of the intervertebral space. The injected cement filled the available intervertebral height. The footprint and height of each PMMA spacer was maximized by injecting 4–6 mL of cement into the available intervertebral space (Figure 1E). Posterior screws were not used for stabilization in line with the aim to investigate the biomechanical behavior and failure of the vertebra–implant interface. Due to the focus being on primary load-bearing capacity without bony ingrowth or fusion, no further filling materials or bone grafts were applied. All CT scans were repeated after treatment (post-op scans, Figure 1D,E).

Trabecular volumetric bone mineral density (BMD) was evaluated from the pre-op CTs using a calibration phantom (B-MAS200, Kyoto Kagaku, Kyoto, Japan) at four axial levels of interest: centrally and in the subcortical regions next to the spacer/cage for both cranial and caudal vertebrae (e-mage Dicom software, V.4.8, Medimon, Hungary, Figure 1B). Following the recommendation of the American College of Radiology [46], vertebrae with an average BMD equal to or higher than 120 mg/cm^3^ were defined as healthy, those with BMD between 80 and 120 mg/cm^3^ as osteopenic and vertebrae with a BMD equal to or lower than 80 mg/cm^3^ as osteoporotic. Further, the central and subcortical cross-sectional areas were calculated from the pre-op CTs together with the anterior and posterior heights of the cranial and caudal vertebrae. Moreover, contact areas, heights and volumes of the PMMA spacers were measured and calculated on the post-op CTs, and the contact area fraction (CAF, spacer contact area over endplate area), contact height fraction (CHF, spacer contact height over vertebral height) and contact volume fraction (CVF, spacer contact volume over vertebral volume) were derived. Spacer contact height was the mean height measured in coronal and sagittal sections of the post-op CT scan within the area of the endplate-spacer contact; spacer contact volume was the product of the mean height and contact area. However, in the case of the PEEK cage, it was impossible to measure the contact areas and heights due to both the concave shape of the vertebral endplates and predominant skew placement of the cage. Therefore, the total area was considered for the calculations, representing the lowest contact stress and best-case scenario for the spacer group. Moreover, the total height and volume of the cage were used. All CT-based evaluations were executed by the same observer using the same method and protocol.

### 2.2. Biomechanical Testing and Data Evaluation

**Figure 2:**
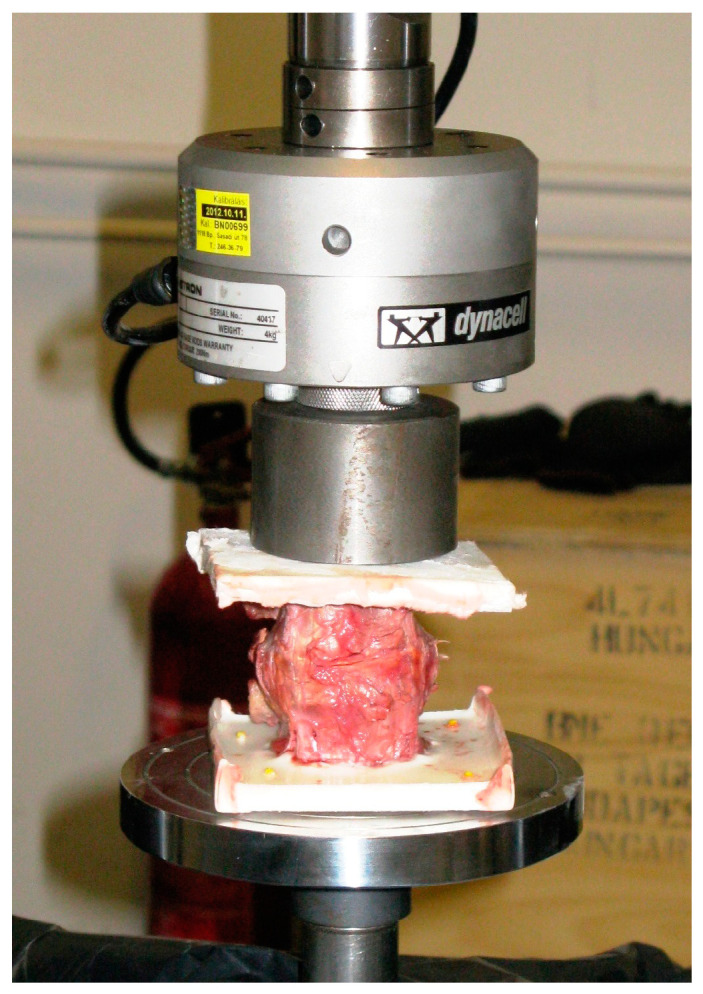
Setup with a specimen mounted for biomechanical testing.

All human cadaveric specimens were stored at −20 °C and thawed at room temperature for 8 h before testing. Quasi-static destructive compression tests were executed in displacement control at a rate of 0.5 mm/min on a servohydraulic testing machine (Instron 8870, Instron, Norwood, MA, USA) equipped with a 25 kN load cell (Figure 2). Destructive monotonic axial compression testing was chosen to focus on the primary mechanical behavior of the vertebra–implant interface in a simple loading mode. Ultimate failure state was defined as either a 20% reduction in reaction force or a 20% specimen deformation. Crosshead displacement and applied force were recorded at 10 Hz.

All mechanical characteristics of the specimens were extracted from the force-displacement curves generated during testing. Stiffness was calculated from the slope of the initial linear portion of the curve. Failure load was represented by the absolute maximum force, and failure displacement corresponded to the respective height loss. Yield strength and yield displacement were indicated by the end of the linear (elastic) part of the curve. Plastic displacement was evaluated by the difference between the failure and yield displacements. Contact failure stress was calculated by dividing the failure load with the contact area of the PEEK or cement spacer.

Statistical analysis was performed using Microsoft Excel v2016. The Independent Samples *t*-test was used to detect significant differences between the groups. Linear regression analysis was performed, and Pearson’s correlation coefficient (R) was used to characterize the correlation between the specimen characteristics and the outcomes of the biomechanical tests. The level of significance was set to 0.05 for all statistical tests.

## 3. Results

### 3.1. Specimen Characteristics

No significant differences were detected between the two treatment groups in terms of donor age, BMD and vertebral geometry (Table 1). Significant differences were detected for contact areas, CAFs, contact volumes and CVFs.

### 3.2. Biomechanical Testing Results

**Figure 3:**
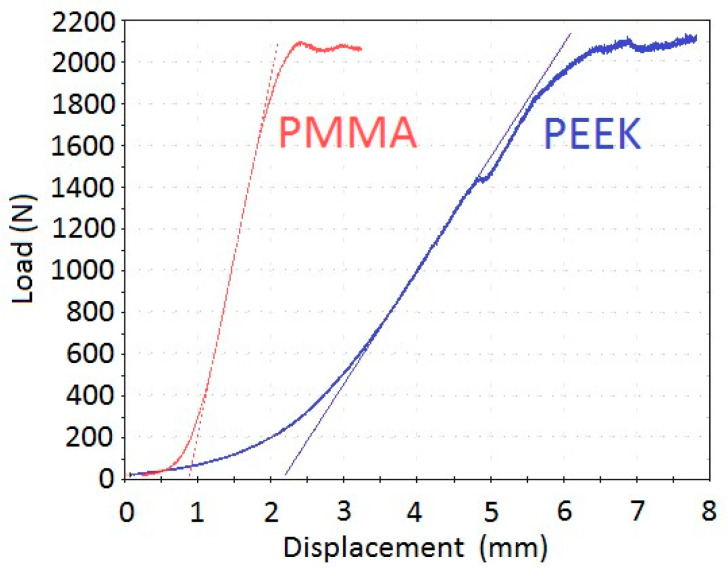
Typical load-displacement curves of specimens treated with PMMA spacers (red) and PEEK cages (blue). Thin lines indicate linear parts for stiffness calculation using the same color code.

The typical load-displacement curves of specimens treated with PEEK cages and PMMA spacers are visualized in Figure 3. Although the failure load was not significantly different between the two treatment groups, stiffness was significantly lower and the failure displacement was significantly larger in the PEEK group (Table 2). The failure load variation was larger in the PEEK group.

### 3.3. Correlation between Specimen Characteristics and Biomechanical Test Results

**Figure 4:**
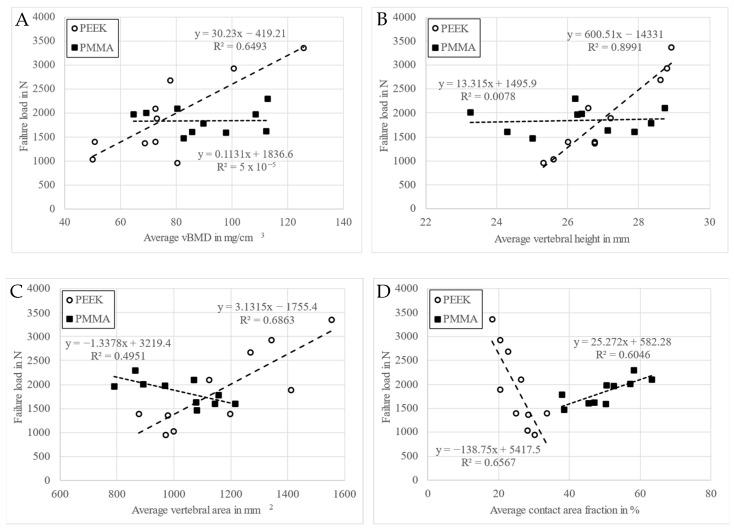
Linear regression plots between failure load and average volumetric bone mineral density (**A**), average vertebral height (**B**), average vertebral area (**C**) and average contact area fraction (**D**) for the two groups, PEEK and PMMA.

Both stiffness and failure load had a strong negative correlation with age and a strong positive correlation with bone density and vertebral height in the PEEK group only (Table 3 and Figure 4A,B). A strong correlation of an opposite sign was found for failure load with vertebral area and CAF for the two groups (Table 3 and Figure 4C,D). For vertebral area, this correlation was strongly positive in the PEEK group and strongly negative in the PMMA group. For CAF, the correlation was strongly negative in the PEEK group and strongly positive in the PMMA group. The linear regression analyses indicated that failure force was higher in the PEEK compared to PMMA group for denser and larger vertebrae, but the opposite was observed for less dense and smaller vertebrae (Figure 4). In addition, the failure load had a strong positive correlation with vertebral volume and a strong negative correlation with CVF in the PEEK group only.

Failure displacement correlated with vertebral anterior height, cranial area and volume positively, and CAF negatively in the PEEK group only. In the PMMA group, failure displacement had a strong negative correlation with CAF and a strong positive correlation with contact height and CHF; and in contrast, stiffness had a strong positive correlation with CAF and a strong negative correlation with contact height and CHF.

### 3.4. Observations: Forms of Failure

**Figure 5:**
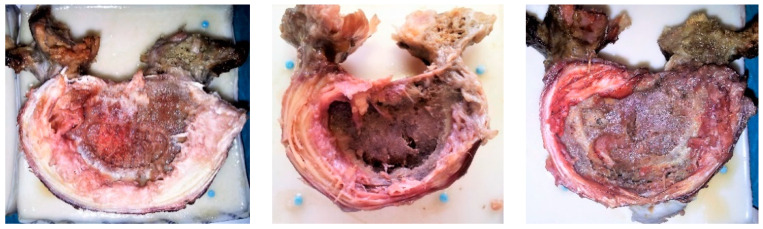
Endplate damage due to PEEK cages illustrating subsidence and fracture.

In the PEEK group, the forms of failure were observed as irreversible spacer subsidence accompanied by endplate fractures along the contact area, mainly for centrally positioned cages (Figure 5). No cage break was observed. In contrast, in the PMMA group, no endplate failures could be visualized since the cement merged with the irregular bone of endplates by solidifying it, and consequently it was not possible to separate them, suggesting that the failure was formed inside the vertebral body.

## 4. Discussion

The injected PMMA cement spacers for lumbar stabilization demonstrated no difference in failure load but significantly higher stiffness and less failure displacement versus traditional D-shaped PEEK cages implanted with the TLIF procedure. These findings were in agreement with previous reports comparing three cage types versus cement spacers used for cervical stabilization [15] or investigating vertebroplasty versus kyphoplasty [47]. The comparable failure load in both treatment groups may have been due to the much stronger materials of the PEEK cages and PMMA spacers than the vertebral bone. The significantly smaller stiffness and higher displacements observed in the PEEK group suggest that, while the total contact area in a segment treated with cement spacer is efficiently utilized in the load transfer from the beginning of the loading process, the load transferring contact area of the cage in the PEEK group, starting from a small value, increased gradually during the compression loading process.

The stiffness and failure load of the specimens with PEEK cages were highly sensitive to BMD, which was in line with an in vitro study demonstrating that vertebral bone density is an effective parameter to predict settling around interbody cages [1], and a review article concluding that axial compressive strength highly depends upon vertebral bone density [12]. A finite element analysis demonstrated that the cage material or loading conditions had a much smaller influence on load-bearing capacity than did cancellous bone density [7]. In another study, stiffness and the ultimate load of cadaveric specimens correlated with the overall vertebral and local subchondral bone densities [2]. Since several reports concluded that the stability of cages in reconstructive surgery depend mostly on the vertebral bone density, severe osteoporosis may be a contraindication for traditional lumbar interbody fusion with cages. On the other hand, our findings for stiffness and the ultimate strength of the specimens with the use of PMMA spacers revealed independence from BMD. We anticipate that this was due to the ability of the cement to accommodate the endplates’ shape, providing a congruent vertebra–implant interface, larger contact area, smoother load transition and less stresses and deformations with higher stiffness, compared to traditional cages.

An experimental study observed penetration of cage spikes into endplates or spongiosa, reporting a strong correlation of stiffness and strength with subchondral BMD, and indicating the importance of endplates’ load-bearing capacity [2]. In the current study, the displacement of specimens with PEEK cages did not correlate with BMD but with anterior vertebral height, indicating that these implants have the greatest subsidence anteriorly due to their anterior-central position following TLIF procedures. Indeed, since both neighboring endplates have concave surfaces, the parallel planes of the PEEK cages contact the endplates mostly anteriorly and locally on a small contact area, and less in the central region of the vertebrae. A previous CT-based bone microstructure analysis highlighted the importance of cage placement on the endplate as well as the vertebral concavity depth on subsidence, stiffness and strength [48]. Biomechanical compression tests on human cadavers concluded that posterolateral placement resulted in higher failure load than central cage placement [3]. In our study, no correlation between displacement and BMD was observed in the PMMA group either, but for a different reason—the injected cement could better accommodate to the irregular endplate, yielding an integral vertebra–implant interface contact, extending through the area of the void created by the nucleus removal. It should be noted that a PMMA spacer is not a classic one. It forms a solid block, a conglomerate with a rather irregular surface, where the cement integrates with the surfaces of the two endplates. That is why we could not show images for the cement spacers, similar to Figure 5, since the removal of the cement from the endplate was not possible. Consequently, an isolated cement spacer could not be produced visually.

The failure load of the specimens with PEEK cages correlated with vertebral area, height and volume, whereas stiffness correlated only with vertebral height. No such correlations were observed in the PMMA group, except for vertebral area. Consequently, failure load was higher in the PEEK versus PMMA group only for denser and larger vertebrae. On the contrary, the correlations indicated that the PMMA spacer could provide higher strength for the clinically more challenging, less dense and/or smaller vertebrae. The correlation of stiffness and strength with vertebral height for the PEEK cages may indicate the important role of the cortical shell in load-bearing situations. In turn, the exclusive correlation of strength with vertebral area for the cement spacer may suggest the diffuse load transfer inside the vertebral body.

The contact area fraction was an important parameter, demonstrating strong negative correlation with failure load in the PEEK group, in contrast to a strong positive correlation in the PMMA group. Assuming that a cage contacts both endplates with its total constant cross-sectional area, the negative correlation with failure load implies that larger vertebrae had higher failure load. The large standard deviation of strength in the PEEK group was probably due to the large variation in contact area. Indeed, the mechanical behavior of a PEEK cage is very sensitive to both its actual footprint [4,6] and actual position [3,8,9,48]. Consequently, the insertion and positioning of the cage into the intervertebral gap can be delicate, especially in the case of osteoporosis. Further, the contact area of the cage increases during the compression loading process and may reach the weaker central part of the endplate, leading to an increasing displacement and/or breaking through the endplate. Since during this process the cage–endplate contact area gradually increases, in the case of endplate damage with a loss in their load capacity, the load transfer may partially shift to other parts of vertebrae and finally to the cortical shell, as indicated by the strong correlation of stiffness and strength with vertebral heights. Thus, the gradually changing contact area can have a critical effect, resulting in a different load-bearing capacity. In turn, the PMMA spacer ensures a larger, congruent contact with endplates remaining constant throughout the loading process, explaining the small scatter of strength. These findings suggest that the location of the cement spacer may be less critical than that of a PEEK cage.

The main benefit of applying cement spacers appears to be their significantly smaller failure displacement compared to the PEEK cages. This seems to be based on the fundamental differences between the two implants regarding the interface with their endplate contact. PEEK cages have a significantly smaller and gradually increasing but still small contact area compared to PMMA spacers. Therefore, the compressive load transfer must be concentrated to this small contact area around the cage teeth, leading to stress concentrations where the cage subsides strongly into the endplates. However, the failure displacement in the PEEK group does not necessarily mean endplate fracture. It is important to distinguish whether the cage only pushes and bends the endplate or breaks through. In the former case, the displacement and the load capacity are affected by the resistance of both the endplate and the spongiosa close to the endplate, while in the case of a fracture, the spongiosa plays a minor role in the subsidence and load capacity—in such a case, the involvement of the cortical shell is crucial. In turn, PMMA spacers provide a significantly larger contact area, and the load transmission is more evenly distributed throughout the whole bone–cement interface without stress risers. Since the cement-covered endplate is always much stronger than the central vertebral spongiosa, the failure is accumulated in the weakest central zone of the vertebrae, leading to diffuse trabecular damage as subsidence. In short, PEEK cages cause a local failure in the endplates with great subsidence, while cement spacers cause a global failure inside the central spongiosa with less subsidence.

In the PMMA group, the strong positive correlation of stiffness and failure load with CAF indicated higher stiffness and strength for specimens with a larger cement covering. In turn, the strong negative correlation of stiffness with the contact cement height and height fractions implies that smaller cement height or higher vertebrae yield larger stiffness. In contrast to the stiffness, the failure displacement in the PMMA group had an opposite correlation sign with CAF and CHF—the higher the cement coverage ratio, the lower the displacement with higher stiffness, and the higher the cement height ratio, the greater the displacement with lower stiffness.

However, in the case of severe osteoporosis, the cement filling can cause excessive stiffness increase in the cement–endplate interface compared to the central part of the vertebral body at a higher risk of central fractures [24]. That is why severe osteoporosis is contraindicated in PCD [23]. At the same time, clinical experience has demonstrated that the incidence of fracture in the case of moderate osteoporosis is not typical, by observing one adjacent fracture among 131 treated levels [25]. A retrospective analysis of patients with 112 lumbar segments with single or multilevel PCD demonstrated that pain and disability significantly decreased [28]. Similarly, clinical analysis after a minimum 1-year follow-up period concluded that for patients with advanced degenerative disc disease and moderate osteoporosis, PCD was an effective minimally invasive treatment option [33].

The different types of failure patterns with PEEK cages or PMMA spacers were demonstrated in a preliminary report using CT-specific finite element simulations, based on the CT data collected in the present study [49]. The authors confirmed that the PEEK cage induced localized plastic deformations primarily in the endplates around the cage, while PMMA spacers accumulated damage mainly in the central trabecular compartment. Consequently, a smaller and a larger trabecular volume may be involved in the resistance with PEEK cages and cement spacers, respectively.

The main limitation of this study was the in vitro nature of the examination, not allowing for either direct extrapolation of the findings to the in vivo reality, or the analysis of the long-term benefits or complications. The results obtained using the twenty motion segments from fifteen human donors may not be applicable to a larger population. However, the donors represent the elderly, predominantly female population affected by the clinical problem, and the samples sizes are in line with standard specimen numbers used in biomechanical research. Only the primary load-bearing capacity could be investigated. Failure was induced via monotonic and quasi-static loading, while in vivo damage is accumulated by repeated activities with lower-force magnitudes. The loading mode, restricted to uniaxial compression, was a simplification of the physiological conditions involving bending. However, in this first biomechanical study on the topic, we aimed at keeping the loading mode simple; more complex loading cases should be used in future studies. A further limitation was that the endplate fracture and subsidence could not be separately evaluated via the machine data of a biomechanical test only. Here, the finite element analysis could provide deeper insights. A further limitation was that posterior implantation was not performed since the aim was to analyze the mechanical behavior of the bone-spacer/cage interface and the damage procedures independently of the effects of screw fixation. However, studies have concluded that posterior instrumentation has no significant effect on the compressive strength of stabilized motion segments [1,12]. Moreover, the injection of PMMA cement in the intervertebral space may be associated with a risk of leakage and related adverse events that were not considered in this biomechanical study performed on extracted motion segments.

## 5. Conclusions

Injected PMMA cement spacer in the lumbar stabilization of osteoporotic spine yielded a similar compression strength, a significantly smaller failure displacement and a stiffer construct than a traditional PEEK cage. While with PEEK cage failure load and stiffness depended strongly on bone density and vertebral geometry, no such relation was observed for cement spacers. PEEK cages imposed a localized load transition with large subsidence into the endplates, but injected PMMA could better accommodate the irregular endplate bone, yielding a massive vertebra–implant interface with smooth load distribution, resulting in diffuse damage in the central spongiosa with smaller subsidence. However, preserving endplate integrity during the insertion of the PEEK cage in osteoporotic cases is critical and often difficult, whereas this is not an issue with the application of a PMMA spacer. PEEK spacers may be more beneficial for larger and denser spines; the cement spacer is less sensitive to density and geometry and therefore could be more suitable for the smaller and less dense vertebrae of elderly patients, helping to decrease spacer subsidence with similar load bearing and enhanced stability in lumbar interbody fixation. In traumatic conditions, the cement spacer is definitely preferable due to its uniform load transfer and independence from bone quality and vertebral geometry. Further studies, mainly case-specific finite element analyses, are required to corroborate these findings and the treatment selection thresholds.

## Figures and Tables

**Table 1:** Age, BMD, vertebral geometry and spacer data for specimens treated with PMMA spacer or PEEK cage in terms of mean value and standard deviation, together with *p*-values from their comparisons between the groups.

Characteristics	Group (*n* = 10)		*p*-Value
	PEEK	PMMA	
Age	70.2 ± 10.6	70.4 ± 13.9	ns
Vertebral BMD (mg/cm^3^)			
mean subcortical	87.4 ± 27.6	98.9 ± 20.3	ns
mean central	67.0 ± 18.0	81.8 ± 16.5	ns
mean cranial	78.9 ± 23.3	93.8 ± 17.6	ns
mean caudal	75.6 ± 21.5	86.8 ± 17.1	ns
mean total	77.2 ± 22.3	90.3 ± 17.2	ns
Vertebral area (mm^2^)			
mean subcortical	1312 ± 259	1143 ± 150	ns
mean central	1032 ± 189	909 ± 138	ns
mean cranial	1145 ± 234	989 ± 138	ns
mean caudal	1199 ± 211	1063 ± 145	ns
mean total	1172 ± 221	1026 ± 140	ns
Vertebral height (mm)			
mean anterior	27.0 ± 1.4	26.7 ± 2.6	ns
mean posterior	27.1 ± 1.6	26.0 ± 1.6	ns
mean cranial	26.8 ± 1.4	26.1 ± 2.2	ns
mean caudal	27.3 ± 1.6	26.6 ± 1.6	ns
mean total	27.1 ± 1.3	26.4 ± 1.9	ns
Vertebral volume (mm^3^)			
cranial	30,989 ± 7770	25,336 ± 4444	ns
caudal	32,881 ± 7202	27,864 ± 5225	ns
mean	31,935 ± 7387	26,600 ± 4780	ns
Spacer contact area ^x^ (mm^2^)	320 ± 0	567 ± 93	****
Contact area fraction ^x^ (CAF, %)			
cranial	25.9 ± 5.4	52.2 ± 9.0	****
caudal	24.6 ± 4.5	48.1 ± 7.8	****
mean	25.2 ± 4.9	50.0 ± 8.2	****
Spacer contact height ^x^ (mm)	10.3 ± 1.5	8.9 ± 2.8	ns
Contact height fraction ^x^ (CHF, %)			
mean anterior	38.1 ± 5.3	33.6 ± 11.6	ns
mean posterior	38.3 ± 6.5	34.2 ± 11.2	ns
mean cranial	38.4 ± 5.2	34.1 ± 11.0	ns
mean caudal	38.0 ± 6.5	33.6 ± 11.8	ns
mean total	38.2 ± 5.9	33.9 ± 11.3	ns
Spacer contact volume ^x^ (mm^3^)	3296 ± 478	4941 ± 1331	**
Contact volume fraction ^x^ (CVF, %)			
cranial	11.1 ± 2.5	20.1 ± 6.9	**
caudal	10.4 ± 2.4	18.3 ± 6.4	**
mean	10.7 ± 2.4	19.2 ± 6.6	**

**: *p* < 0.01, ****: *p* < 0.0001, ns: non-significant; ^x^: maximum values for PEEK cages, considering total contact; real values for PMMA spacers.

**Table 2:** Results from biomechanical testing for specimens treated with PMMA spacers or PEEK cages in terms of mean value and standard deviation, together with *p*-values from their comparisons between the groups.

Outcome	Group (*n* = 10)		*p*-Value
	PEEK	PMMA	
Stiffness (N/mm)	525 ± 181	1192 ± 376	****
Failure load (N)	1915 ± 836	1847 ± 267	ns
Failure displacement (mm)	5.64 ± 1.05	3.16 ± 0.81	****
Yield strength (N)	1636 ± 770	1483 ± 320	ns
Yield displacement (mm)	4.74 ± 0.98	2.38 ± 0.44	****
Plastic displacement before failure (mm)	0.90 ± 0.56	0.79 ± 0.59	ns
Failure contact stress ^x^ (N/mm^2^)	5.98 ± 2.61	3.32 ± 0.64	**

**: *p* < 0.01, ****: *p* < 0.0001, ns: non-significant; ^x^: minimum value for PEEK cages, considering total contact; real value for PMMA spacers.

**Table 3:** Correlation coefficients (R) of specimen characteristics with stiffness, failure load and failure displacement for specimens treated with PEEK cage or PMMA spacer.

Correlation	Stiffness				Failure Load				Failure Displacement			
	PEEK		PMMA		PEEK		PMMA		PEEK		PMMA	
	R	P	R	P	R	P	R	P	R	P	R	P
Age	−0.791	*	ns		−0.922	***	ns		ns		ns	
BMD												
mean subcortical	0.672	*	ns		0.803	*	ns		ns		ns	
mean central	0.732	*	ns		0.763	*	ns		ns		ns	
mean cranial	0.705	*	ns		0.821	*	ns		ns		ns	
mean caudal	0.712	*	ns		0.784	*	ns		ns		ns	
mean total	0.712	*	ns		0.806	*	ns		ns		ns	
Vertebral area												
mean subcortical	ns		ns		0.849	**	−0.707	*	ns		ns	
mean central	ns		ns		0.774	*	−0.663	*	ns		ns	
mean cranial	ns		ns		0.842	**	−0.740	*	0.625	*	ns	
mean	ns		ns		0.803	**	−0.661	*	ns		ns	
mean total	ns		ns		0.828	**	−0.704	*	ns		ns	
Vertebral height												
mean anterior	ns		ns		0.844	**	ns		0.712	*	ns	
mean posterior	0.877	**	ns		0.833	**	ns		ns		ns	
mean cranial	0.677	*	ns		0.871	**	ns		ns		ns	
mean caudal	0.752	*	ns		0.819	*	ns		ns		ns	
mean total	0.807	*	ns		0.948	****	ns		ns		ns	
Vertebral volume												
cranial	ns		ns		0.792	*	ns		0.623	*	ns	
caudal	ns		ns		0.879	**	ns		ns		ns	
mean	ns		ns		0.882	**	ns		ns		ns	
Spacer contact area ^x^			ns				ns				ns	
Contact area fraction ^x^												
cranial	ns		ns		−0.844	**	0.821	*	−0.619	*	ns	
caudal	ns		0.682	*	−0.762	*	0.717	*	ns		−0.682	*
mean	ns		0.660	*	−0.810	*	0.778	*	ns		−0.645	*
Spacer contact height ^x^	ns		−0.625	*	ns		ns		ns		0.720	*
Contact height fraction ^x^												
anterior	ns		−0.620	*	ns		ns		ns		0.655	*
posterior	ns		−0.638	*	ns		ns		ns		0.712	*
cranial	ns		−0.631	*	ns		ns		ns		0.659	*
caudal	ns		−0.629	*	ns		ns		ns		0.708	*
mean	ns		−0.631	*	ns		ns		ns		0.687	*
segment	ns		−0.638	*	ns		ns		ns		0.689	*
Spacer contact volume ^x^	ns		ns		ns		ns		ns		ns	
Contact volume fraction ^x^												
cranial	ns				−0.827	**	ns		ns			
caudal	ns		ns		−0.716	*	ns		ns		ns	
mean	ns		ns		−0.780	*	ns		ns		ns	
segment	ns		ns		−0.780	*	ns		ns		ns	

*: *p* ≤ 0.05, **: *p* < 0.01, ***: *p* < 0.001, ****: *p* < 0.0001, ns: non-significant; ^x^: Nominal values for PEEK cages considering total contact, real values for PMMA spacers.

## Data Availability

The data presented in this study are available on request from the corresponding author. The data are not publicly available due to confidentiality reasons.
